# Activity limitation and social participation restriction among leprosy patients in Boru Meda Hospital, Amhara Region, Ethiopia

**DOI:** 10.1371/journal.pntd.0008702

**Published:** 2020-09-24

**Authors:** Seid Getahun Abdela, Saskia van Henten, Seid Hassen Abegaz, Fentaw Bialfew Bayuh, Feleke Tilahun Zewdu, Fentaw Tadese Berhe, Johan van Griensven

**Affiliations:** 1 Department of internal medicine, College of Medicine and Health sciences, Wollo University, Dessie, Ethiopia; 2 Unit of neglected tropical diseases, Institute of Tropical Medicine, Antwerp, Belgium; 3 Dermatology unit, Boru Meda Hospital, Dessie, Ethiopia; 4 School of Public Health, College of Medicine and Health sciences, Wollo University, Dessie, Ethiopia; Universidade Federal do Para, BRAZIL

## Abstract

**Background:**

Although Ethiopia eliminated leprosy as public health problem 20 years ago, still more than 3000 new cases are reported annually. Leprosy related disability affects patients’ day to day physical activities and their participation in social activities. Assessing the degree of activity limitation and social participation is recommended to show disability and assess the efficacy of rehabilitation efforts.

**Methodology and principal finding:**

A hospital based cross sectional study was conducted among a total of 305 leprosy patients. Data were collected by face to face interview using Screening of Activity Limitation and Safety Awareness (SALSA) scale and participation scale. The analysis was done with SPSS version 25. Descriptive statistics was done and then binary logistic regression was used to identify factors associated with activity limitation as well as participation limitation.

Most patients (219, 71.8%) had activity limitation; 41 (13.4%) with severe and 25 (8.2%) with extreme limitations. More than half of patients (168, 55.1%) were suffering from participation restriction; with 43 (14.1%) having severe restriction and 30 (9.8%) extreme restriction. Older age, low educational status, distance from treatment center, time of treatment and higher Eye, Hand, Foot disability score were associated with activity limitation. Similarly, older age, low educational status and being unmarried were significantly associated with participation restriction.

**Conclusion:**

This study revealed that activity limitation and participation restriction are common among leprosy patients. Earlier diagnosis and improved rehabilitative services may help to decrease activity limitation, whereas community rehabilitation may improve social participation. The old and centralized leprosy rehabilitation services need to be decentralized and backed with modern equipment and trained staffs.

## Author summary

To show a complete picture of disability, combining different disability assessment tools is recommended. For the first time in Ethiopia, we described the degree of activity and social participation limitation of adults with leprosy attending one of the leprosy centers. We used internationally validated measurement tools, the Screening Activity Limitation Safety Awareness score (SALSA score) for activity limitation and the participation scale (P-scale) for assessment of social participation. Twenty years after elimination of leprosy as a public health problem, leprosy patients continue to suffer from different forms of disabilities. Most patients suffered from activity limitation and social participation restriction. Our finding indicates the unmet need of disability prevention and management. We stress here the importance of public health efforts to diagnose and manage the disease early and to work on rehabilitation of leprosy patients. The old and centralized leprosy rehabilitation services need to be decentralized and backed with modern equipment and trained staffs.

## Introduction

Leprosy is a chronic infectious disease that mainly affects the skin and peripheral nerves, leading to neuropathy and related complications, including disability and physical disfigurement. The disease is historically related with stigma, particularly in the presence of visible deformity. Despite elimination of leprosy as a public health problem in 2000 both globally and at the national level, new cases continue to appear [1]. Over 200,000 new leprosy cases were reported in 2018 [2].

Ethiopia is one of the countries within sub-Saharan Africa with the highest leprosy case load. The leprosy elimination target of less than one case per 10,000 populations has been reached at national level in 1999. However, the new case notification remains the same for the past ten years ranging between 3000–4000 new cases per year. It is among the countries reporting the highest number of new leprosy cases with grade two disability [3]. In a recent study, a total of 57 new patients presented in a three-month period, of which almost 60% had grade II disability, and 89% were multibacillary(MB) [4].

If multi-drug treatment is initiated early, it can cure leprosy and prevent disability. However, late diagnosis continues to be a reason for patients presenting at hospital with irreversible leprosy complications [5]. Leprosy related disabilities affect different domains of human life. The physical disabilities make day to day activities difficult and affect their income. The disease may cause community and family devaluation of affected persons leading to psychosocial problems [6], with resulting stigma leading to discrimination and social exclusion. Both physical and psychosocial problems can severely affect quality of life in leprosy patients [7].

To avert the potential consequences of disabilities owing from leprosy complications, community based rehabilitation program (CBR) and institution-based rehabilitation are recommended for management of leprosy patients with disabilities [6,8]. However, these services are not well organized, donor dependent and centralized only in five leprosy centers in Ethiopia. Leprosy associations are in charge of coordinating CBR for several years with no or minimal government involvement. Currently, they are facing different challenges including lack of sufficient funding. For these reasons, they are not recruiting newly diagnosed cases. Institution based physical rehabilitation is also dependent on non-governmental organizations and is only available in the five-leprosy centers (including Boru Meda Hospital). However, even in those five leprosy centers the service often suffers from lack of trained staffs and necessary equipment.

Despite WHO’s recommendation of routine assessment of social participation for CBR efficacy evaluation, it was not done for both program and research purposes in Ethiopia [9]. So, assessing participation restriction is urgently needed to identify CBR related gaps for improvement of the programs. Additionally, social participation restriction is directly related to stigma, and therefore, a better understanding of the level of social participation and associated factors will help to plan different stigma reduction strategies like empowering victims and promoting inclusions. To obtain a full picture of disability, activity limitation should also be measured. Understanding the degree of activity limitation is an important parameter in assessing the efficacy of current institution based rehabilitative programs. Therefore, in this study we assessed activity limitation and participation restriction and their associated factors among leprosy patients in the Boru Meda Hospital leprosy center of Ethiopia.

## Methods

### Study setting

Boru Meda Hospital is one of the five leprosy centers in Ethiopia which was founded in 1954. The hospital was initially established by missionaries mainly for treating those with leprosy and its complications, including ophthalmologic ones. Currently, up to 1000 leprosy patients visit the hospital for diagnosis, follow-up, complication treatment and rehabilitation annually.

The management of leprosy and its complications follows the national and WHO guideline. Diagnosis of new cases is mainly clinical, complemented with microscopic evaluation of skin samples. Previously diagnosed and treated leprosy cases visit the hospital for different complications. For management of leprosy-related ophthalmic complications, organized ophthalmologic services with two ophthalmologists and five optometrists were available. Reconstructive surgery for injuries caused by nerve damage was started by an orthopedic surgeon in 2018. Recently, a rehabilitative center was established within the hospital but it is currently not functional as it not supported with enough professionals and equipment.

### Study design, population and sample size

Adult leprosy patients with current or previous diagnosis of leprosy visiting Boru Meda Hospital between June 2019 and January 2020 were invited to participate in this cross-sectional study. This included newly diagnosed patients, cases currently on treatment and patients who finished chemotherapy coming for disability management. They were recruited from both the outpatient (OPD) and inpatient department. Patients with co-morbidities which can affect activity and social participation, like mental illness, retroviral infection and diabetes mellitus were excluded.

The sample size was calculated using Epi Info 7; using the sample size formula for the estimation of a single proportion. The settings for the sample size calculation were: expected proportion of significant participation restriction of 50%, 95% confidence level and precision of 5%. After correction for total expected patient flow of 990 patients and additional 10% non-response rate, the final sample size was 305.

### Data collection procedures

After obtaining verbal informed consent, study participants were interviewed using a structured questionnaire that included socio-demographic and clinical information (leprosy classification, type of reaction and disability grading) and an evaluation of social participation and activity limitation. In addition, the medical charts of patients were reviewed to record the presence as well as type of of leprosy reactions.

Cases were categorized into paucibacillary (PB) (≤5 skin lesions and/or only one affected nerve trunk) or MB leprosy (>5 skin lesions and/or >1 affected nerve trunk) using the WHO leprosy classification. Leprosy reactions were defined as episodes characterized by acute inflammation of skin lesions or nerves (type 1) and/or the appearance of inflamed cutaneous nodules with/without neuritis (type 2) [1]. EHF (Eye, Hand and Feet) score (sum of the six components of the WHO impairment grading) was used for grading of impairment [10].

The SALSA (Screening Activity Limitation Safety Awareness) scale was used to measure the activity restriction component of disability. The scale was designed to be used for people with peripheral neuropathy. The score ranges from 0 to 80, with the following recommended cutoffs/categories: no significant limitation (0–24), mild limitation (25–39), moderate limitation (40–49), severe limitation (50–59) and extreme limitation (60–80) [11,12]. We used the P scale (Participation scale) which incorporates the participation domains of the International Classification of Functioning, Disability and Health of the WHO to measure perceived participation limitations. It has 18 items and the resulting P-score ranges from 0 to 90. A P-Score higher than 12 points is considered a significant restriction with the need for social rehabilitation. The score from 0–12 signifies no significant restriction, 13–22 mild restriction, and 23–32 moderate restriction, 33–52 severe restriction and 53–90 extreme restriction [13].

The questionnaires were filled by trained interviewers in a quiet private room. Training was given for two days using P scale and SALSA scale manuals [14,15]. When a respondent did not understand the meaning of a question, the interviewer re-read the question and did not explain the sentence with other words. The interviewers were not involved in the treatment of patients. During interview respondents were provided adequate time for recall when it was necessary.

### Data management and analysis

Data was entered using Epi-data and then exported to SPSS version 25 for further analysis. Summary statistics like mean, median and, frequency and proportions were computed to describe socio-demographic and clinical profile variables. Activity limitation and social participation restriction were dichotomized as being present or absent. Activity limitation was labeled as present when SALSA score >24 and absent when the SALSA score was ≤24. Participation restriction was labeled as present when P score>12 and absent when the P score ≤ 12 as no restriction. Binary logistic regression models were fitted (for activity limitation and social participation restriction separately) to identify factors associated with activity limitation and social participation restriction. First bivariate models were made and variables with p-value less than 0.2 were considered as candidate for the final multivariable regression analysis. Afterwards, all candidate variables were added to the multivariable model and variables having a p-value of less than 0.05 and adjusted odds ratio (AOR) with 95% Confidence Interval (CI) non inclusive of one were considered as significant predictors of significant activity limitation as well as participation restriction.

### Ethical considerations

Participation in the study was voluntary. Consent was sought from each participant followed by a written informed consent. Permission for the study was secured from Wollo University, College of Medicine and Health Sciences ethics committee. The study was carried out according to the principles stated in the Declaration of Helsinki, all applicable regulations and according to established international scientific standards.

## Results

### Socio-demographic characteristics

All 305 invited leprosy patients were included in to the study making a 100% response rate. The majority (243,79.7%) of patients were males and the mean (standard deviation) age of the study participants was 45 (±15.84) years. Most patients (224, 73.4%) had no formal education and they were from the rural areas (235, 77%), married (211, 69.2%) and farmers (207, 67.9%) (Table 1).

**Table 1:** Socio demographic characteristics.

Variables	Frequency	Percentage
**Sex**		
Male	243	79.7
Female	62	20.3
**Age**		
18–35	88	28.9
36–50	111	36.4
51–65	72	23.6
>65	34	11.1
**Educational Status**		
No formal education	224	73.4
Primary education	50	16.4
Secondary Education	24	7.9
Tertiary Education	7	2.3
**Marital Status**		
Married	211	69.2
Single	57	18.7
Divorced	25	8.2
Widowed	12	3.9
**Residence**		
Rural	235	77
Urban	70	23
**Occupational status**		
Farmer	207	67.9
Unemployed	55	18.0
Merchant	18	5.9
Governmental employee	16	5.2
Beggar	6	2.0
Others	3	1.0

### Clinical profile

The majority (282, 92.5%) of patients were seen and managed at the outpatient department. One fourth (77, 25.2%) of them were newly diagnosed. Most study participants were classified as MB cases (297, 97.4%). Leprosy reactions were documented for 131 (43%) cases; of which,48 (36.6%) had type 1 and 83 (63.4%) had type 2 reactions. The median EHF score was 4 (IQR: 1–7).

Most cases (286; 93.8%) were treated after the introduction of multidrug therapy, whereas 19 (6.7%) received monotherapy. The majority of patients (213, 69.8%) received chemotherapy only, while the others (92, 30.2%) received physiotherapy and/or reconstructive surgery (Table 2).

**Table 2:** Clinical profile.

Variables	Frequency	Percentage
**Time since treatment**		
Newly diagnosed (not yet on treatment)	77	25.2
Currently on treatment	23	7.5
Finished treatment in the last one year	27	8.9
Finished treatment (1-5years)	39	12.8
Finished treatment (5-10years)	35	11.5
Finished treatment before 10 years	104	34.1
**Site of care**		
OPD	282	92.5
Ward	23	7.5
**WHO type**		
Multibacillary	297	97.4
Pauci-bacillary	8	2.6
**Leprosy reactions**		
No reaction	174	57.0
Current or previous reactions	131	43
**Type of leprosy reaction (n = 131)**		
Type 1	48	36.6
Type 2	83	63.4
**Type of treatment**		
Multidrug	286	93.8
Monotherapy	19	6.2
**Additional measures given**		
Yes	92	30.2
No	213	69.8
**Additional measures given (n = 92)**		
Physiotherapy	61	66.3
Reconstructive surgery	7	7.6
Both physiotherapy and reconstructive surgery	24	26.1
**EHF score**		
< 5	168	55.1
≥ 5	137	44.9

OPD: outpatient department, EHF: Eye Hand Feet, WHO: World Health Organization

### Activity limitation and participation restriction

The mean (SD) SALSA score was 36.9 (±15.2). Most of the patients (219, 71.8%) had activity limitation; of whom 41 (13.4%) with severe and 25 (8.2%) with extreme limitations. The mean (SD) P scale was 20.1 (±21.2). Most of the patients (168; 55.1%) were suffering from some degree of participation restriction; of whom 43 (14.1%) had severe restriction and 30 (9.8%) had extreme restriction (Table 3).

**Table 3:** Activity limitation and participation restriction.

Variable/Categories	Frequency	Percentage
**Levels of SALSA score**		
No significant limitation	86	28.2
Mild limitation	104	34.1
Moderate limitation	49	16.1
Severe limitation	41	13.4
Extreme limitation	25	8.2
**Levels of P scale score**		
No significant restriction	137	44.9
Mild restriction	51	16.7
Moderate restriction	44	14.4
Severe restriction	43	14.1
Extreme restriction	30	9.8

SALSA: Screening Activity Limitation Safety Awareness, P Scale: Participating scale

### Factors associated with activity limitation

Several factors were found to be associated with significant activity limitation. Patients with a high EHF score, having no formal education, living further from the hospital, being older than 45 years and having finished treatment more than a year ago significantly increased the odds of activity limitation.

Patients above 45 years were 2.7 times more likely to have significant activity limitation compared to participants below 45 years (AOR = 2.72, 95% CI: 1.35–5.47). The odds of significant activity limitation were 3.6 times higher among leprosy patients who had no formal education compared to patients with secondary and above level of education (AOR = 3.63, 95% CI: 1.33–9.90). Patients with total EHF score greater than or equal to five had more than 12 times higher odds of significant activity limitation compared to patients with total EHF score less than five (AOR = 12.57, 95% CI: 5.25–30.09). Patients who finished treatment were 1.96 times more likely to develop significant activity limitation than newly diagnosed and currently on treatment groups (AOR = 1.96, 95% CI: 1.04–3.70). Patients who traveled more than 10 kilometers from the treatment center were 2.86 times more likely to have significant activity limitation compared to those closer to the hospital (AOR = 2.86, 95% CI: 1.21–6.77) (Table 4).

**Table 4:** Factors associated with activity limitation.

Variables	SALSA score (Activity limitation)		Crude odds ratio (95% CI)	Adjusted odds ratio (95% CI)
	Yes	No		
**Age (years)**				
> 45	132	17	6.16 (3.39–1.17)	**2.72 (1.35–5.47)**
≤ 45	87	69	1	1
**Educational status**				
No formal education	178	46	8.13 (3.58–8.45)	**3.63 (1.33–9.90)**
Primary school	31	19	3.43 (1.33–8.82)	2.39 (0.77–7.36)
Secondary and above	10	21	1	1
**Distance from treatment center**^**a**^				
>10 km	189	66	1.91 (1.01–3.59)	**2.86 (1.21–6.77)**
≤10 km	30	20	1	1
**Occupation**				
Farmer and beggars	161	52	1.82 (1.07–3.07)	0.77 (0.35–1.71)
Other occupation	58	34	1	1
**Residence**				
Rural	177	58	2.03 (1.16–3.57)	1.37 (0.58–3.21)
Urban	42	28	1	1
**Time of treatment**				
Previously treated	162	43	2.84 (1.69–4.78)	**1.96 (1.04–3.70)**
Newly diagnosed/on treatment	57	43	1	1
**Lepra reaction**				
Reaction present	107	24	2.46 (1.44–4.24)	1.31 (0.66–2.62)
No reaction	112	62	1	1
**EHF score**				
≥ 5	130	7	16.49 (7.27–37.38)	**12.57 (5.25–30.09)**
< 5	89	79	1	1

^a^ Calculated for the district which the patient came from

EHF: Eye, Hand and Feet, SALSA: Screening Activity Limitation Safety Awareness; CI: Confidence Interval

### Factors associated with participation restriction

For patients above 45 years, the odds of significant participation restriction were two times higher than patients aged less than or equal to 45 years (AOR = 1.99, 95% CI: 1.11–3.56). Leprosy patients who had no formal education or primary level of education had 4.76 and 4.51 times higher odds of significant participation restriction compared to those patients with secondary and above respectively (AOR = 4.76, 95% CI: 1.54–14.67) and (AOR = 4.51 CI: 1.36–14.97). Those leprosy patients with an EHF total score greater than or equal to five had 5.97 times higher odds of significant participation restriction compared to patients with EHF total score less than five (AOR = 5.97 CI: 3.56–10.61). Similarly, participants with single or divorced or widowed marital status were associated with 2.04 times higher odds of significant participation restriction compared to married participants (AOR = 2.04, 95%CI: 1.06–3.93) (Table 5).

**Table 5:** Factors associated with participation restriction.

Variables	P scale (Participation restriction)		Crude odds ratio	Adjusted odds ratio
	Yes	No		
**Age**				
> 45	105	44	3.52 (2.19–5.67)	**1.99 (1.11–3.56)**
≤ 45	63	93	1	1
**Educational status**				
No formal education	136	88	6.44 (2.54–16.33)	**4.76 (1.54–14.67)**
Primary school	26	24	4.5 (1.58–12.89)	**4.51 (1.36–14.97)**
Secondary and above	6	25	1	1
**Marital status**				
Single/Divorced/Widowed	60	34	1.68 (1.02–2.77)	**2.04 (1.06–3.93)**
Married	108	103	1	1
**Residence**				
Rural	136	99	1.63 (0.95–2.79)	1.25 (0.64–2.44)
Urban	32	38	1	1
**Occupation**				
Farmer and beggars	124	89	1.52 (0.93–2.48)	1.05 (0.51–2.17)
Others occupation	44	48		1
**Time of treatment**				
Previously treated	126	79	2.20 (1.35–3.58)	1.23 (0.67–2.24)
Newly diagnosed/on treatment	42	58	1	1
**Lepra reaction**				
Reaction present	81	44	2.27 (1.42–3.63)	1.61 (0.93–2.81)
No reaction	87	93	1	
**EHFscore**				
≥ 5	112	25	8.96 (5.23–15.37)	**5.97 (3.56–10.61)**
< 5	56	112	1	1

EHF: Eye, Hand and Feet

## Discussion

For the first time in Ethiopia, this study assessed leprosy patients’ activity limitation and social participation restriction using internationally validated scales. Our findings are alarming, with more than two-thirds of leprosy patients suffering from limited activities and more than half restricted from social participations. Several factors were found to be associated with significant activity limitation. Patients with a high EHF score, having no formal education, living further from the hospital, being older than 45 years and having finished treatment more than a year ago significantly increased the odds of activity limitation. Correspondingly, patients with a high EHF score, having no formal education or primary school, being older than 45 years and being married were significantly and positively associated with significant participation restriction.

More than 70% of patients had activity limitation and this reveals the severity of nerve damage and related disability. This result is much higher than findings from Bangladesh and Brazil [16,17]. This could be explained by difference in leprosy patients’ profile, including timing of diagnosis and difference in disability care. A high proportion of patient with activity limitation reflects late diagnosis and management and lacking or inadequate physical rehabilitation. Efforts for early diagnosis should get priority and different ways to reinforce and decentralize leprosy case detection should be explored. Health extension workers could play a role in awareness raising, case detection and referral. Likewise, strengthening the barely existing physical rehabilitation programs should get more emphasis. Rehabilitation for leprosy patients may be extended beyond the five leprosy centers. Specialists involvement in diagnosis, monitoring and management of leprosy related impairment could improve physical rehabilitation [18].

The proportion of patients with participation restriction was 55.1% which is higher than findings from Indonesia and Brazil and lower than the result from Nigeria[19–21]. This might be variation in study participants level of education and other socio-demographic and economic variables. The limitations in participation may be caused by several factors including self-stigmatization, activity limitation, family related issues and poverty and low level of education and the community low awareness level that stigmatize due to a fear of contagion. Moreover, the problem is also likely to be related to insufficient rehabilitation services including community-based rehabilitation programs. Community-based rehabilitation programs along with other disability prevention strategies were found to improve social participation [19]. Integrating CBR in existing vertical leprosy programs and monitoring using WHO CBR indicators [9] could be implemented. New case diagnosis and management may be systematically linked with CBR programs.

Age was found to be significantly associated with activity limitation as well as participation restriction. The age and activity limitation association is similar with the finding from Brazil [20]. This could be explained by age related activity limitation. Patients who finished treatment were more likely to have significant activity limitation compared to patients who are newly diagnosed or on treatment. This could be explained by better rehabilitation services including reconstructive surgery, which was started in 2018 [16]. Like other studies, low educational level and high EHF score were associated with both participation restriction and activity limitation [22]. Patients with higher educational level may be more knowledgeable about leprosy symptoms and could have better health seeking behavior. This might result in early diagnosis of leprosy, thereby reducing disability and related problems. Efforts on physical disability prevention and management could improve physical activities and social engagement of leprosy patients.

Distance from the treatment center was associated with significant activity limitation. Those near to the hospital may have early diagnosis and management which resulted in better functionality. Furthermore, difficult transport access, unable to afford transport cost, and poor access to information may result in poor health seeking behavior and delayed diagnosis. Being single, divorced and widowed were also associated with significant social participation restriction. Couples in which one person is affected by leprosy could have discussions and share ideas about felt stigma and psychosocial problems, which could be important coping mechanisms.

Although leprosy has officially been eliminated as a public health problem, patients still suffer significantly due to leprosy complications. Unfortunately, the declaration of elimination has led to a decrease in funding for leprosy programs, which has led to a suboptimal functioning of several rehabilitation programs which could have decreased stigma and improved functioning of leprosy patients.

The study has some limitations. First, it is difficult to make causal association as the study design is cross-sectional. Second, as the study lacks healthy individuals as control group; it is difficult to assess whether findings are only disease-related. Finally, the study is prone for selection bias as the study is hospital-based.

This study revealed that, activity limitation and participation restriction are common among leprosy patients. Earlier diagnosis and improved rehabilitative services may help to decrease activity limitation, whereas community rehabilitation may improve participation. Leprosy patients of old age and those with no formal education need more attention. Both institution and community-based rehabilitation gaps should be identified and addressed. Regular monitoring and evaluation of these programs is needed. The old and centralized leprosy rehabilitation service need to be decentralized and backed with modern equipment and trained staffs.

## Supporting information

S1 File Leprosy Final (2). (SAV)
